# Elevated expression of thyroid hormone receptor **α** 2 (c-erb A- **α** 2) in nasopharyngeal carcinoma[Fn tlnote1]

**DOI:** 10.1054/bjoc.2000.1505

**Published:** 2000-12

**Authors:** J-W Lee, C-L Chen, B-T Juang, J-Y Chen, C-S Yang, S-L Doong

**Affiliations:** Graduate Institute of Microbiology, College of Medicine, National Taiwan University, Taipei, Taiwan; Department of Pathology, National Taiwan University Hospital, Taipei, Taiwan

**Keywords:** nasopharyngeal carcinoma, c-erb A-α2 (TR-α2), differential display, RNA in situ hybridization

## Abstract

Differential display was used to identify genes differentially expressed between cultured normal nasal epithelial cells and nasopharyngeal carcinoma (NPC) cell lines. A 130 bp cDNA fragment showing homology with thyroid hormone receptor α2 (TR-α2 or c-erb A-α2) was identified in NPC cell lines. Northern blot analysis using the 130 bp cDNA fragment and a TR-α2 specific cDNA containing part of the coding region as probes, we were able to detect a 2.7 kb transcript corresponding to that of TR-α2 in NPC cell lines but not in normal nasal epithelial cells. RNA in situ hybridization was used to detect TR-α2 expression in clinical biopsies obtained from NPC patients and non-tumour controls. TR-α2 mRNA was detected in 1 out of 24 (4.2%) normal nasopharynx epithelium biopsies, in 5 out of 27 (18.5%) primary and 15 out of 24 (62.5%) recurrent tumours. The positive rate of TR-α2 expression in recurrent NPC biopsies was significantly higher than that in normal nasopharynx epithelium (*P*  < 0.00001). The relevance of the elevated expression of TR-α2 in the pathogenesis process of NPC was discussed. © 2000 Cancer Research Campaign http://www.bjcancer.com


*British Journal of Cancer* (2000) **83**(12), 1674–1680
© 2000 Cancer Research Campaign
doi: 10.1054/ bjoc.2000.1505, available online at http://www.idealibrary.com on IDEAL®

http://www.bjcancer.com

### Elevated expression of thyroid hormone receptor α2 (c-erb A-α2) in nasopharyngeal carcinoma*

**J-W Lee[1], C-L Chen[2], B-T Juang[1], J-Y Chen[1], C-S Yang[1] and S-L Doong[1]**

[1]Graduate Institute of Microbiology, College of Medicine, National Taiwan University, Taipei, Taiwan; [2]Department of Pathology, National Taiwan University Hospital, Taipei, Taiwan

**Summary** Differential display was used to identify genes differentially expressed between cultured normal nasal epithelial cells and nasopharyngeal carcinoma (NPC) cell lines. A 130 bp cDNA fragment showing homology with thyroid hormone receptor α2 (TR-α2 or c-erb A-α2) was identified in NPC cell lines. Northern blot analysis using the 130 bp cDNA fragment and a TR-α2 specific cDNA containing part of the coding region as probes, we were able to detect a 2.7 kb transcript corresponding to that of TR-α2 in NPC cell lines but not in normal nasal epithelial cells. RNA in situ hybridization was used to detect TR-α2 expression in clinical biopsies obtained from NPC patients and non-tumour controls. TR-α2 mRNA was detected in 1 out of 24 (4.2%) normal nasopharynx epithelium biopsies, in 5 out of 27 (18.5%) primary and 15 out of 24 (62.5%) recurrent tumours. The positive rate of TR-α2 expression in recurrent NPC biopsies was significantly higher than that in normal nasopharynx epithelium ($P < 0.00001$). The relevance of the elevated expression of TR-α2 in the pathogenesis process of NPC was discussed. © 2000 Cancer Research Campaign http://www.bjcancer.com

**Keywords**: nasopharyngeal carcinoma; c-erb A-α2 (TR-α2); differential display; RNA in situ hybridization

Nasopharyngeal carcinoma (NPC) is an endemic cancer with a very high incidence in South China and Southeast Asia such as Hong-Kong and Taiwan. Epstein-Barr virus (EBV), genetic factors (human HLA type A2-BW42 and cytochrome p4502E1-CYP2E1), and certain dietary (nitrosamine, herbal medicine) and environmental conditions (smoking, occupational exposures) have been shown to be involved in the development of NPC (Hildesheim and Levine, 1993), but the molecular mechanisms by which NPC was generated remained largely unknown. Over-expression of Myc oncogene was detected in NPC cell lines (Lin et al, 1993) and in some NPC tumours (Porter et al, 1994). Bcl-2 was up-regulated and was detected in most samples of NPC (Lu et al, 1993). Mutations in p53 tumour suppressor gene (Spruck et al, 1992) and RB/p105 gene (Sun et al, 1993) in NPC are relatively rare. Lately, mutations in the RB2/p130 gene were detected in 30% of primary human NPCs (Claudio et al, 2000). Though decreased level of expression was reported, no point mutation of the potential tumour suppressor gene p16 was detected in NPC (Sun et al, 1995). These studies suggested the importance of other genes in NPC oncogenesis.

Various tools such as cytogenetic analysis (Mitelman et al, 1983; Huang et al, 1989), loss of heterozygosity (LOH) (Huang et al, 1991; Hu et al, 1996; Mutirangura et al, 1997), and comparative genomic hybridization (CGH) (Chen et al, 1999; Hui et al, 1999) have been employed to investigate the candidate genes associated with NPC. These studies offered a genome-wide investigation based upon a comprehensive pattern of DNA sequence copy number changes. Alterations due to mutations and/or regulation dysfunction can hardly be detected. Differential display (DD) (Fung et al, 1998) and cDNA representational difference analysis (RDA) (Zhan et al, 1998) offered alternative screening tools. We had adopted differential display for the study of differences in gene-expression patterns between normal nasal epithelial and transformed nasopharyngeal epithelial cells. The elevated expression of TR-α2 in NPC cell lines was demonstrated. RNA in situ hybridization of TR-α2 transcripts among biopsies derived from NPC cases and control subjects was employed to substantiate the in vitro finding.

### MATERIALS AND METHODS

### Cell culture

Biopsied tissues of nasal polyps and turbinates were collected from National Taiwan University Hospital and Mackay Memorial Hospital, Taipei. After removal of blood clots with three washes of sterile phosphate buffered saline (PBS), these samples were added into Dulbecco's minimum essential medium (DMEM) supplemented with 0.1 mM nonessential amino acids, 50 U ml$^{-1}$ penicillin, 50 μg ml$^{-1}$ streptomycin, 1.25 μg ml$^{-1}$ fungizone and 1 mg ml$^{-1}$ pronase. After an overnight incubation at 4°C, carcass was discarded and the suspension was centrifuged at 1,500 rpm for 10 min at room temperature. The pelleted cells were resuspended in DMEM supplemented with 10% fetal bovine serum, 0.1 mM nonessential amino acids, 50 U ml$^{-1}$ penicillin, 50 μg ml$^{-1}$ streptomycin, 1.25 μg ml$^{-1}$ fungizone, and inoculated onto collagen coated plates. Attached cells were grown to 70–80% confluence at 37°C and served as normal epithelial cells control. Cytokeratin staining was employed to verify the nature of the attached cells. More than 95% of the attached cells showed positive cytokeratin

*Received 25 February 2000*
*Revised 3 July 2000*
*Accepted 10 August 2000*

*Correspondence to:* S-L Doong

*Supported in part by National Science Council (NSC 87-2314-B-002-268, NSC 88-2314-B-002-153) and the National Health Research Institute (DOH83-H-315, DOH84-H-315, DOH85-H-315)

staining, demonstrating epithelial cells origin. NPC cell lines, HONE-1 (Glaser et al, 1989), NPC-TW01 (Lin et al, 1990) and NPC-TW04 (Lin et al, 1993), were all grown at the same condition. LCL, EBV-immortalized lymphoblastoid cells, was kindly provided by Dr Chin-Hwa Tsai at the Graduate Institute of Microbiology, National Taiwan University.

### Differential display (DD-PCR)

Total RNAs were extracted from cells grown at 70–80% confluence according to the method described by Chomczynski and Sacchi (Chomczynski and Sacchi, 1987). 50 micrograms of total RNAs were treated with 10 units of RNase free DNase, extracted with phenol/$CHCl_3$, and precipitated with ethanol. The reverse transcription and PCR reaction were performed as described by manufacturer's instruction (RNAimage Kit, GenHunter Corporation). Briefly, reverse transcription was done in the reaction mixtures containing 2 μg total RNAs, 50 mM Tris, pH 8.3, 3 mM $MgCl_2$, 75 mM KCl, 10 mM DTT, 0.02 mM each dNTP, 2.5 μM H-$T_{11}$N (5′-AAGCTTTTTTTTTTTN-3′, N = A, G, or C), 20 U RNase inhibitor, and 200 U MMLV-RT (Life Technologies) in a volume of 20 μl at 35˚C for 1 h. After the reaction, the whole mixture was heated to 94˚C for 5 min, followed by dilution of the reverse transcription reaction mixture to 200 μl. Two microlitres of the dilution mixture was combined with 20 μl reaction mixture containing 10 mM Tris-HCl, pH 8.8, 1.5 mM $MgCl_2$, 50 mM KCl, 0.1% Triton-X100, 2 μM each dNTP, 2.5 μM H-$T_{11}$N, 0.5 μM arbitrary 13 mers (H-AP1 to H-AP80), 2.5 U Taq polymerase (DYNAZYME, Finnzymes Inc, Finland), 10 μCi α-$^{35}$S-dATP (> 1000 Ci $mmol^{-1}$, Amersham Pharmacia Biotech) for PCR. The PCR products were separated in a 6% DNA sequencing gel and visualized by autoradiography. Bands of interest were cut out from the polyacrylamide gel, and the eluted and reamplified cDNA fragments were cloned into the *Eco*RV-cut and T-tagged pBluescript SK vector (Stratagene). For each fragment, 6 to 10 colonies containing inserts of correct size were chosen for sequence analysis and homology search with nucleic acid data bank.

### Northern analysis

Poly-A(+) RNA were purified through affinity chromatography on oligo-(dT)-cellulose (Life Technologies). 20 μg of total RNAs and 5 μg of poly-A(+) RNA were size-fractionated on 1% agarose gel containing formaldehyde, blotted on Hybond-N™ membrane (Amersham Pharmacia Biotech). The blot was hybridized with $^{32}$P-labelled probes and exposed to phosphor-screen (Molecular Dynamics) and scanned using PhosphoImager (STORM 840, Molecular Dynamics). The same blots were stripped and reprobed with glyceraldehyde-3-phosphate dehydrogenase (GAPDH) as an internal control.

### Southern blot analysis

20 micrograms of total DNAs extracted from NPC cell lines or LCL were digested with *Eco* RI, *Hind* III, *Pst* I or *Xho* I, separately. DNA fragments were separated in a 1% agarose gel electrophoresis and transferred to Hybond-N™ membrane. The membrane was hybridized with $^{32}$P-labelled rat TR-α1 cDNA fragment and visualized by PhosphoImager (Molecular Dynamics).

### Plasmid and cRNA probe preparation

EST clone (IMAGE clone Id 42706), containing nt. 356–2015 of TR-α2 cDNA (gene bank accession no. j03239) was purchased from Genome Systems, Inc, sequenced and matched to TR-α2 cDNA as reported. An approximately 600 bp (nt. 1410–2015) DNA fragment, covering the unique region of TR-α2, was isolated after *Eco*RI digestion and ligated into *Eco*RI-cut pBluescript SK vector, generating pTRα2CSK. The fragment was inserted in an orientation so that sense strand can be transcribed by T3 RNA polymerase and anti-sense strand can be transcribed by T7 RNA polymerase. pTRα2CSK was linearized with *Bam*HI or *Hind*III and served as template for antisense or sense riboprobe synthesis, respectively. The riboprobe synthesis was performed in a 20 μl reaction containing 1 μg DNA template, 1 mM ATP, CTP, and GTP, 0.65 mM UTP, 0.35 mM digoxigenin-11-UTP, 50 units of RNase inhibitors, 40 units of T3 or T7 RNA polymerase (Boehringer Mannheim) in transcription buffer at 37˚C for 1 hour. The reaction was terminated by incubation with 2 units of RNase-free DNase I (Life Technologies) for 15 minutes at 37˚C. The digoxigenin-labelled probe was precipitated with ethanol and dissolved in DEPC-treated water.

### Specimens

The present work comprised 27 primary NPC patients and 24 radio- or chemotherapy treated and recurrent NPC patients. Nasopharyngeal tissues from patients with chronic inflammation but without evidence of tumours were used as source of normal nasopharyngeal epithelium. The histological diagnosis of all patients was confirmed by pathologist. Histopathological classification of all cases was performed according to WHO classification. All specimens were received from pathology file of National Taiwan University Hospital and fixed in 10% neutralized formalin and embedded in paraffin.

### RNA in situ hybridization

5 μm of paraffin-embedded sections were deparaffinized in xylene and hydrated to 50% ethanol then dried, after which they were treated with prewarmed 20 μg $ml^{-1}$ proteinase K for 10 min at 50˚C, washed with water. The slides were dipped into freshly prepared 0.25% acetic anhydride in 0.1 M triethanolamine, pH 8.0, for 10 minutes, washed with phosphate buffered saline (PBS), DEPC-treated water, then dried. The sections were incubated with a final concentration of 0.5 ng $ml^{-1}$ denatured digoxigenin-labelled sense or antisense riboprobes in hybridization buffer (50% formamide, 5 × SSC, 10% dextran sulphate, 2% SDS, 1% PVP, 5 × Denhardt's solution, 100 μg $ml^{-1}$ denatured salmon sperm DNA) at 42˚C for 16–18 hours. The sections were then washed in 1 × SSC and 0.2 × SSC/0.01% SDS at room temperature for 5 min, followed by washing in 0.05 × SSC at 50˚C for 5 min, and 0.025 × SSC at 55˚C for 15 min. After advanced incubation in buffer 1 (100 mM Tris-HCl, 150 mM NaCl, pH 7.5), sections were blocked with 2% blocking reagent (Boehringer Mannheim) for 1 hour at room temperature, then washed in buffer 1. Anti-digoxigenin antibody conjugated with alkaline phosphatase at 1:200 dilution in buffer 1 containing 1% swine serum was applied onto the sections for 1 hour at room temperature. Subsequently, the sections were washed in buffer 1 and buffer 2 (100 mM Tris-HCl, 100 mM NaCl, 50 mM $MgCl_2$, pH 9.5) for 5 min at room temperature,

*© 2000 Cancer Research Campaign*

respectively. Finally, in situ signal was developed with NBT/BCIP solution (Boehringer Mannheim) and nuclear fast red was utilized as a counterstain reagent. Two-tailed, Fisher's exact test was used for statistical analysis, and a *P* value of less than 0.05 was graded as statistically significant.

### RESULTS

NPC, also termed as 'lymphoepithelioma', is characterized by the close association of lymphoid cells and transformed epithelial cells. To avoid the complication of the heterogeneous cell composition of clinical NPC specimens, we used the established cultured NPC cells: HONE-1 (Glaser et al, 1989), NPC-TW01 (Lin et al, 1990), and NPC-TW04 (Lin et al, 1993) for the DD assay. Due to the difficulty in collecting a critical mass of normal epithelial cells from nasopharynx, primary epithelial cells cultured from polyps and turbinates were utilized as normal controls. Although they were derived from different anatomical sites, they were all characterized as pseudostratified columnar respiratory epithelial cells.

In this study, a total of 144 primer pair combinations were finished and 597 cDNA fragments were collected. Of these, 54 cDNA fragments were tested in the Northern blot analysis and 16 were confirmed to be differentially expressed. Results of homology search using the Blast program were listed in Table 1. Six of these sequences were found to show significant homology with EST clones without known function. The remainder matched to the following sequences: cadherin-6, caldesmon, laminin, glutamin synthetase, tropomyosin, c-erb A-α2, cyclin B, lactate dehydrogenase B, cyclin B2, and PTI-1. The 130 bp cDNA fragment (designated $T_{11}CAP_2NPCl$-8), identified in all three NPC cell lines (Figure 1A), showed 98% homology with the 3′-untranslated region of human TR-α2 (c-erb A-α2) (Nakai et al, 1988) (Figure 1B). When utilized as a probe, a 2.7 kb transcript was detected in all three NPC cell lines (Figure 2A, lanes 1–3) but not in normal control obtained from a randomly-picked individual (Figure 2A, lane 4) or pooled samples (Figure 2A, lane 5). In order to examine whether TR-α2 (c-erb A-α2) was indeed differentially expressed, the poly A+ RNAs were extracted and probed with a cDNA fragment (nts. 1410–2015) of TR-α2 (IMAGE clone Id 42706). As expected, the 2.7 kb transcript was exclusively detected in HONE-1 cells (Figure 2B, lane 1) but not in normal control (Figure 2B, lane 2).

TR-α2 gene has been mapped to chromosome 17q11.2. Significant chromosome gains at 17q in NPC biopsy specimens have been found using comparative genomic hybridization (CGH) (Chen et al, 1999). DNA amplification induced-elevation in TR-α2 expression level was suspected. Southern blot analysis was

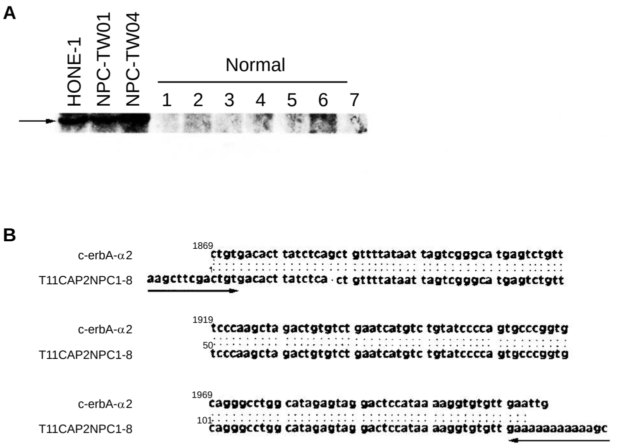

**Figure 1** Band pattern and sequence alignment of $T_{11}CAP_2NPC1$-8 cDNA fragment with c-erb A-α2. (**A**) Band pattern from a part of the sequencing gel in differential display of mRNAs from three NPC cell lines: HONE-1, NPC-TW01, NPC-TW04 and cultured normal nasal epithelial cells derived from 7 individual. Arrow indicates $T_{11}CAP_2NPC1$-8 cDNA that is expressed in all three NPC cell lines but not in normal cultured epithelial cells. (**B**) Sequence alignment of $T_{11}CAP_2NPC1$-8 cDNA fragment with c-erb A-α2. Sequences of primers employed to generate the cDNA fragment are underlined. The numbering of c-erb A-α2 sequences is based upon that reported by Nakai *et al*. (1988)

**Table 1** Results of homology search of the 16 differentially expressed cDNA fragments

| Source derived from | cDNA fragment | Fragment size (bp) | Homology search | Size of transcript[a] |
|---|---|---|---|---|
| Cultured normal nasal epithelial cells | $T_{11}GAP_5N5c$-8 | 400 | Cadherin-6 | 8 kb |
| | | | | 4 kb |
| | $T_{11}CALtk_3CN3$ | 379 | AA610677[b] | 3.5 kb |
| | $T_{11}GAP_5N5b$ | 550 | Caldesmon | 2 kb |
| | | | | 5 kb |
| | $T_{11}GAP_6N6c$ | 350 | laminin | 5 kb |
| | $T_{11}GAP_6N6d$-19 | 280 | AW614173[b] | 4.8 kb |
| | $T_{11}AAP_2N2d$-1 | 420 | Glutamin synthetase | 3 kb |
| | $T_{11}AAP_8N8a$-3 | 620 | Tropomyosin isoform mRNA | 2 kb |
| NPC cell lines | $T_{11}CAP_2NPC1$-8 | 130 | c-erb A-α2 (TR-α2) | 2.7 kb |
| | $T_{11}GAP_2NPC2d$ | 500 | N48003[b] | 4 kb |
| | $T_{11}GAP_2NPC2e$ | 400 | AA282043[b] | 1 kb |
| | $T_{11}GAP_3NPC3d$ | 500 | Cyclin B | 1.8 kb |
| | $T_{11}GAP_7NPC7j$-4 | 260 | AA340599[b] | 2 kb |
| | | | | 5 kb |
| | $T_{11}CAP_9NPC9b$-5 | 420 | Lactate dehydrogenase | 1 kb |
| | $T_{11}CAP_{10}NPC10b$-1 | 700 | Cyclin B2 | 2 kb |
| | $T_{11}CAP_{11}NPC11c$ | 450 | AA411599[b] | 2 kb |
| | $T_{11}CAP_{13}NPC13a$ | 350 | PTI-I | 4.8 kb |
| | | | | 2.1 kb |
| | | | | 0.5 kb |

[a]size estimated by Northern blot analysis using $^{32}$P-labelled cDNA fragment designated in the same row. [b]EST clone.

 *© 2000 Cancer Research Campaign*

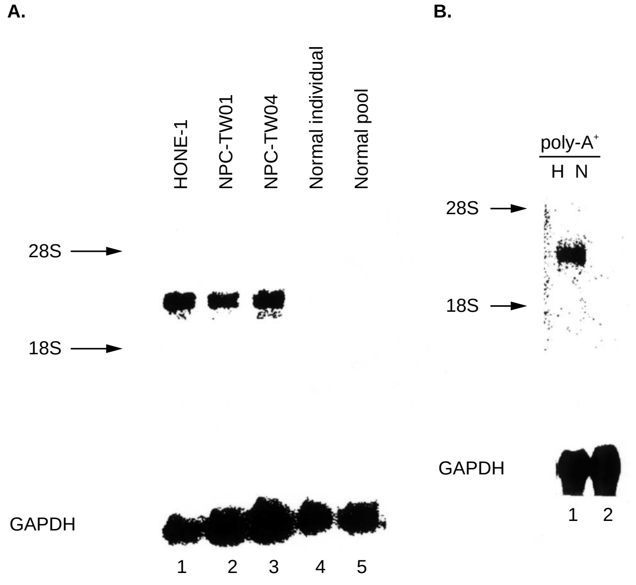

**Figure 2** Northern profiles of c-erb A-α2 (TR-α2) gene specifically expressed in NPC cell lines. (**A**) 20 micrograms of total RNAs from NPC cell lines: HONE-1 (lane 1), NPC-TW01 (lane 2), NPC-TW04 (lane 3), cultured epithelial cells of polyps from a normal individual (lane 4) and pooled controls (lane 5) were loaded into indicated lanes of 1% agarose gel containing formaldehyde. A 2.7 kb transcript was detected in all three NPC cell lines when probed with $^{32}$P-end-labelled cDNA fragment ($T_{11}CAP_2NPC1$-8) obtained from DD-PCR cloning. The same blot was reprobed with GAPDH as an internal control and shown at the bottom. (**B**) Five micrograms of poly-A(+) RNAs from H: HONE-1 (lane 1) or N: cultured normal epithelial cells (lane 2) were loaded into 1% agarose containing formaldehyde. The 2.7 kb transcript was detected in HONE-1 cell but not in cultured normal epithelial cells when probed with $^{32}$P-labelled cDNA fragment containing the unique region (nts 1410 to 2015) of c-erb A-α2 (TR-α2) gene. The same blot was reprobed with GAPDH as an internal control and shown at the bottom

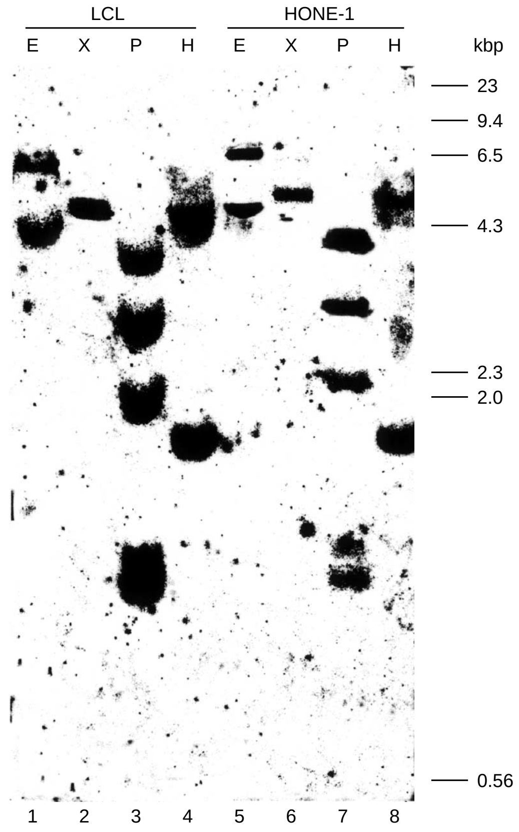

**Figure 3** Southern blot analysis revealing similar TR-α genomic organization between HONE-1 and LCL cell lines. 20 micrograms of DNA extracted from LCL (lanes 1-4) or HONE-1 cells (lanes 5–8) were digested with *Eco* RI (E), *Xho* I (X), *Pst* I (P) or *Hind* III (H) separately, and loaded into 1% agarose gel. After electrophoresis, the separated DNA fragments were transferred to Hybond-N$^{TM}$ membrane and hybridized with $^{32}$P-labelled TR-α cDNA probes performed to investigate the genomic organization of TR-α gene in NPC cell lines. No amplification or obvious DNA rearrangement was observed (Figure 3).

Due to the complication of heterogeneous cell population in biopsies, RNA in situ hybridization, instead of Northern blot analysis or RT-PCR, was utilized to examine the status of TR-α2 expression. The present study included biopsies obtained from 27 patients with primary NPC, 24 patients with recurrent NPC, and 24 patients with chronic inflammation but without evidence of neoplasm. The clinical and pathological features are listed in Table 2. 22 (81.5%) of the primary and 19 (79.2%) of the recurrent tumours were classified as undifferentiated carcinoma (WHO type III), 5 (18.5%) of the primary and 4 (16.7%) of the recurrent tumours were non-keratinizing carcinoma (WHO type II). The sex distribution was 20 (74.1%) males, 7 (25.9%) females in the primary tumours, and 20 (83.3%) males, 4 (16.7%) females in the recurrent tumours. Sense or anti-sense riboprobes covering the unique region (nts. 1410–2015) of TR-α2 were used for RNA in situ hybridization. When more than 10% of the epithelial cells were stained purple-red, it was scored as positive (Figure 4). The results are summarized in Table 2. One of 24 (4.2%) normal nasopharyngeal specimens, 5 of 27 (18.5%) primary NPC tumours, and 15 of 24 (62.5%) recurrent tumours exhibited positive staining. Expression of TR-α2 was not correlated with histological type or clinical stage of tumours. The difference between normal controls and the recurrent NPC tumours was statistically significant ($P < 0.00001$, two-tailed Fisher's exact test). A significant difference was observed between recurrent and primary NPC tumours ($P = 0.0018$). The difference in frequency between normal controls (4.2%) and the primary NPC tumours (18.5%) was statistically not significant ($P = 0.106$), possibly due to the limitation of sample size.

### DISCUSSION

We have applied the mRNA differential display method to identify genes differentially expressed in cultured NPC or normal nasal epithelial cells. Among the 597 fragments collected, 54 were tested in Northern blot analysis and 16 were confirmed to be differentially expressed (Table 1). Several distinct sequences have been identified by Fung et al (1998) using the same approach. Representational difference analysis employed by Zhan et al (1998) revealed several sequences too. However, not a single sequence was identical among these two and our studies, possibly due to the utilization of different cell culture conditions or primer sets used.

Among those 16 listed in Table 1, TR-α2 attracted us most. Thyroid hormone receptors (TRs) are members of nuclear hormone receptors. Nuclear hormone receptors, such as oestrogen receptor (ER), androgen receptor (AR), have been implicated in the outgrowth of tumour cells. Abnormal nuclear receptors are involved in a variety of cancers, including human acute promyelocytic leukaemia, which is caused by chromosomal translocations resulting in fusion of RARα to other cellular proteins (Warrell

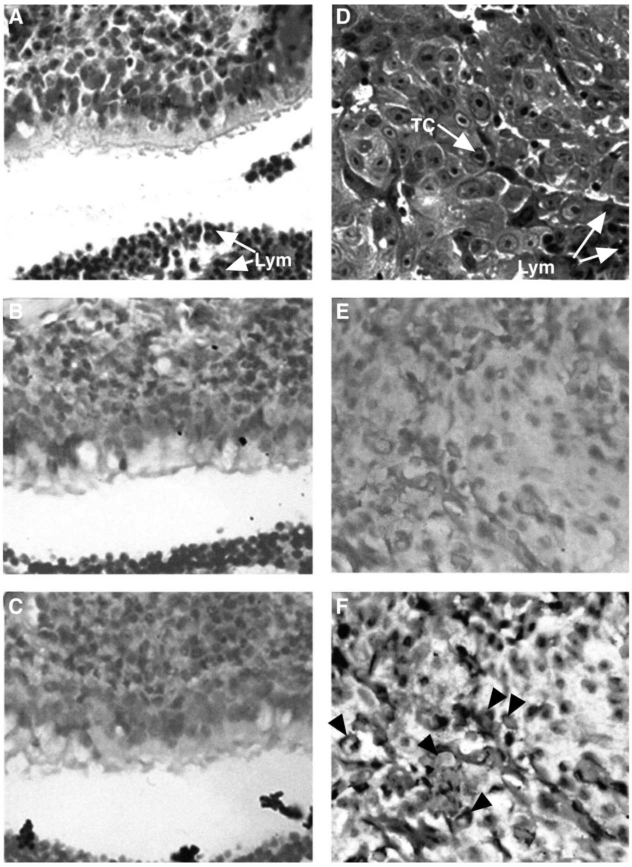

**Figure 4** RNA in situ hybridization showing c-erb A-α2 (TR-α2) expression in NPC but not in normal nasopharynx epithelium. Five µm of paraffin-embedded normal nasopharynx epithelium (**A,B,C**), and NPC tumour (**D,E,F**) sections were applied with digoxigenin-11-UTP labelled sense (**B,E**) or antisense (**C,F**) riboprobes of TR-α2 gene and developed with NBT/BCIP solution. Nuclear fast red was used as counterstain reagent. ➨ indicated positive staining. HE staining (A,D) of sections are shown in the first row. NPE: normal nasopharynx epithelium, Lym: infiltrated lymphocytes, TC: nasopharyngeal carcinoma cells. Original magnification × 360

et al, 1994). TR-α2 (c-erb A-α2) is an alternatively spliced variant of the TR-α1 (c-erb A-α1) gene (Lazar et al, 1988; Mitsuhashi et al, 1988). TR-α2 (c-erb A-α2) is unable to bind T3 and has been shown to exert dominant negative action of T3/TRs-dependent transactivation of target genes (Koenig et al, 1989; Lazar et al, 1989). This dominant negative effect is similar to that observed for the oncoprotein v-erb A, whose C-terminal mutations render it unable to bind T3 (Damm, 1993). The dominant negative v-erb A contributes to the development of erythroblastic leukaemia after infection with avian erythoblastosis virus. Hepatocellular carcinoma was observed in male mice transgenic for v-erb A (Barlow et al, 1994), demonstrating that v-erb A can promote neoplasia in mammals. By analogy with its dominant negative action on T3/TRs-dependent transcription to v-erb A, TR-α2 (c-erb A-α2) might contribute certain roles in the unstrained growth phenotype. In fact, increased expression of c-erb A-α2 mRNA was reported in transformed rodent cells when compared with their non-transformed counterparts (Too and Guernsey, 1992). Lately, the high prevalence of TR mutations was reported in tumour cells of hepatocellular carcinoma, suggesting that the mutant TRs could play an important role in liver carcinogenesis (Lin et al, 1999). The evidence for direct linkage of abnormal TR-α2 expression with human neoplasm is absent. This is the first report describing the association of elevated TR-α2 expression with nasopharyngeal carcinoma. Studies are in process to determine whether the elevated expression of TR-α2 in NPC cells did provide the cells with a selective growth advantage.

Southern blot analysis indicated that there was no amplification or obvious genomic organization change of TR-α gene (Figure 3) in NPC cell lines. The exact mechanism by which TR-α2 expression level was elevated in NPC cell lines remains to be elucidated. Small genomic lesions such as mutations, deletions awaits detailed sequencing analysis.

RNA in situ hybridization revealed a moderate increase in percentage of positive TR-α2 expression in primary NPC specimens (18.5%) compared to that in normal control (4.2%). Though the difference was statistically not significant, the number and intensity of positive TR-α2 staining in primary NPC specimens were consistently higher and stronger than normal control. Whether elevated expression of TR-α2 is causally related to or merely a consequence of the development of NPC requires further examination. The result that merely one-fifth of the primary NPC specimens demonstrated TR-α2 expression implies the involvement of other genes in the development of NPC.

The finding that a high percentage of biopsies derived from recurrent NPC tumours were scored positive (15 in 24) for TR-α2 expression is interesting. Most NPC cases are initially treated with radiotherapy or chemotherapy, cancer recurrence usually implies that tumour cells have escaped from cytotoxic effects of radiation or chemotherapy. While the result offered evidence for increased

 *© 2000 Cancer Research Campaign*

**Table 2** Clinical data and in situ hybridization results of TR-α2 in patients with NPC

| Case number | Sex | Age | WHO type | Stage | Primary | Recurrent |
|---|---|---|---|---|---|---|
| 1[a] | M | 46 | II | IV | – | |
| 2 | M | 46 | III | IV | +[b] | |
| 3 | M | 38 | III | | + | |
| 4 | M | 43 | III | III | – | |
| 5 | M | 47 | III | II | – | |
| 6 | M | 41 | III | | + | |
| 7 | M | 34 | III | II | – | |
| 8 | M | 42 | III | II | + | |
| 9 | M | 58 | II | II | – | |
| 10 | M | 36 | II | IV | – | |
| 11 | M | 59 | II | IV | – | |
| 12 | M | 38 | III | | – | |
| 13 | M | 53 | III | II | – | |
| 14 | M | 29 | III | II | + | |
| 15 | M | 32 | III | | – | |
| 16 | M | 31 | III | III | – | |
| 17 | M | 44 | III | IV | – | |
| 18 | F | 56 | III | III | – | |
| 19 | F | 44 | III | II | – | |
| 20 | F | 35 | III | II | – | |
| 21 | F | 47 | III | | – | |
| 22 | M | 31 | II | II | – | – |
| 23 | F | 56 | III | | – | – |
| 24 | F | 45 | III | | – | – |
| 25 | M | 49 | III | | – | + |
| 26 | M | 43 | III | III | – | + |
| 27 | F | 25 | III | II | – | + |
| 28 | M | 44 | III | | | – |
| 29 | M | 54 | II | | | + |
| 30 | M | 43 | III | | | + |
| 31 | M | 37 | II | | | + |
| 32 | M | 35 | III | | | – |
| 33 | M | 43 | III | | | + |
| 34 | M | 31 | III | | | + |
| 35 | M | 67 | III | | | + |
| 36 | M | 50 | III | | | + |
| 37 | M | 51 | III | | | + |
| 38 | M | 62 | III | | | + |
| 39 | M | 67 | II | | | + |
| 40 | M | 57 | III | | | – |
| 41 | M | 43 | III | | | + |
| 42 | M | 47 | III | | | + |
| 43 | M | 47 | III | | | – |
| 44 | M | 60 | I | | | – |
| 45 | F | 68 | III | | | – |

[a]Cases 1–27 are primary tumours; cases 22–45 are recurrent tumours.[b]A positive was scored when more than 10% of epithelial cells were stained purple-red.

tolerance to cytotoxic challenge in NPC cells with TR-α2 expression, the possibility of radio-/chemotherapy-induced changes and subsequently elevated expression level of TR-α2 can not be excluded.

In addition to studies based on DNA/chromosome alterations, study such as this RNA-based screening method offered an alter native and supplementary way to identify putative NPC-related oncogenes or tumour suppressor genes. The current study using RNA-based differential display in conjugation with clinical biopsy studies revealed TR-α2 as a candidate gene and provided a feasible approach for the search of the NPC-related molecules.

### ACKNOWLEDGEMENTS

We are greatly indebted to the Department of Otolaryngology of Mackay Memorial Hospital and Dr Te-Huei Yeh of the National Taiwan University Hospital for collecting the nasal polyps and turbinates. We would like to thank Dr Hey-Chi Hsu (Department of Pathology, National Taiwan University) and Dr Shuang-En Chuang (National Health Research Institute) for discussion and critical reading of this manuscript.

### REFERENCES

Barlow C, Meister B, Lardelli M, Lendahl U and Vennstrom B (1994) Thyroid abnormalitites and hepatocellular carcinoma in mice transgenic for v-erb A. *EMBO J* **13**: 4241–4250

Chen YJ, Ko JY, Chen PJ, Shu CH, Hsu MT, Tsai SF and Lin CH (1999) Chromosomal aberrations in nasopharyngeal carcinoma analyzed by comparative genomic hybridization. *Genes Chromosomes Cancer* **25**: 169–175

Chomczynski P and Sacchi N (1987) Single-step method of RNA isolation by acid guanidinium thiocyanate-phenol-chloroform extraction. *Anal Biochem* **162**: 156–159

© 2000 Cancer Research Campaign

Claudio PP, Howard CM, Fu Y, Cinti C, Califano L, Micheli P, Mercer EW, Caputi M and Giordano A (2000) Mutations in the retinoblastoma-related gene RB2/p130 in primary nasopharyngeal carcinoma. *Cancer Res* **60**: 8–12

Damm K (1993) Erb A: tumor suppressor turned oncogene? *FASEB J* **7**: 904–909

Fearon ER and Vogelstein B (1990) A genetic model for colorectal tumorigenesis. *Cell* **61**: 759–767

Fung LF, Yuen PW, Wei W, Kwan HS and Tsao SW (1998) Identification of genes differentially expressed in nasopharyngeal carcinoma by messenger RNA differential display. *Int J Oncol* **13**: 85–89

Glaser R, Zhang HY, Yao KT, Zhu HC, Wang FX, Li GY, Wen DS and Li YP (1989) Two epithelial tumor cell lines (HNE-1 and HONE-1) latently infected with Epstein-Barr virus that derived from nasopharyngeal carcinoma. *Proc Natl Acad Sci USA* **86**: 9524–9528

Hildesheim A and Levine PH (1993) Etiology of nasopharyngeal carcinoma: a review. *Epidemiologic Rev* **15**: 466–485

Hu LF, Eiriksdottir G, Lebedeva T, Kholodniouk I, Alimov A, Chen F, Luo Y, Zabarovsky ER, Ingvarsson S, Klein G and Ernberg I (1996) Loss of heterozygosity on chromosome arm 3p in nasopharyngeal carcinoma. *Genes Chromosomes Cancer* **17**: 118–126

Huang DP, Ho JH, Chan W-K, Lau W-H and Liu M (1989) Cytogenetics of undifferentiated nasopharyngeal carcinoma xenografts from southern Chinese. *Int J Cancer* **43**: 936–939

Huang DP, Lo K-W, Choi HK, Ng YT, Tsao S-Y, Yiu KC and Lee CK (1991) Loss of heterozygosity on the short arm of chromosome 3 in nasopharyngeal carcinoma. *Cancer Genet Cytogenet* **51**: 91–99

Hui AB-Y, Lo K-W, Leung S-F, Teo P, Fung MKF, To KF, Wong N, Choi PHK, Lee JCK and Huang DP (1999) Detection of recurrent chromosomal gains and losses in primary nasopharyngeal carcinoma by comparative genomic hybridisation. *Intl J Cancer* **82**: 498–503

Koenig RJ, Lazar MA, Hodin RA, Brent GA, Larsen PR, Chin WW and Moore DD (1989) Inhibition of thyroid hormone action by a non-hormone binding c-erbA protein generated by alternative mRNA splicing. *Nature (London)* **337**: 659–661

Lazar MA, Hodin RA, Darling DS and Chin WW (1988) Identification of a rat c-erbA alpha-related protein which binds deoxyribonucleic acid but does not bind thyroid hormone. *Mol Endocrinol* **2**: 893–901

Lazar MA, Hodin RA and Chin WW (1989) Human carboxyl-terminal variant of alpha-type c-erbA inhibits trans-activation by thyroid hormone receptors without binding thyroid hormone. *Proc Natl Acad Sci USA* **86**: 7771–7774

Lin C-T, Wong C-I, Chan W-Y, Tzung K-W, Ho K-C, Hsu M-M and Chuang S-M (1990) Establishment and characterization of two nasopharyngeal carcinoma cell lines. *Lab Invest* **62**: 713–724

Lin C-T, Chan W-Y, Chen W, Huang H-M, Wu H-C, Hsu M-M, Chuang S-M and Wang C-C (1993) Characterization of seven newly established nasopharyngeal carcinoma cell lines. *Lab Invest* **68**: 716–727

Lin K-H, Shieh H-Y, Chen S-L and Hsu H-C (1999) Expression of mutant thyroid hormone nuclear receptors in human hepatocellular carcinoma cells. *Mol Carcinog* **26**: 53–61

Lu QL, Elia G, Lucas S and Thomas JA (1993) bcl-2 proto-oncogene expression in Epstein-Barr-virus-associated nasopharyngeal carcinoma. *Int J Cancer* **53**: 29–35

Mitleman F, Mark-Vendel E, Mineur A, Giovanella B and Klein G (1983) A 3q+ marker chromosome in EBV-carrying nasopharyngeal carcinomas. *Int J Cancer* **32**: 651–655

Mitsuhashi T, Tennyson GE and Nikodem VM (1988) Alternative splicing generates messages encoding rat c-erbA proteins that do not bind thyroid hormone. *Proc Natl Acad Sci USA* **85**: 5804–5808

Mutirangura A, Tanunyutthawongese C, Pornthanakasem W, Kerekhanjanarong V, Sriuranpong V, Yenrudi S, Supiyaphun P and Voravud N (1997) Genomic alterations in nasopharyngeal carcinoma: loss of heterozygosity and Epstein-Barr virus infection. *Br J Cancer* **76**: 770–776

Nakai A, Seino S, Sakurai A, Szilak I, Bell GI and DeGroot LJ (1988) Characterization of a thyroid hormone receptor expression in human kidney and other tissues. *Proc Natl Acad Sci USA* **85**: 2781–2785

Porter MJ, Field JK, Leung SF, Lo D, Lee JC, Spandidos DA and van Hasselt CA (1994) The detection of the c-myc and ras oncogenes in nasopharyngeal carcinoma by immunohistochemistry. *Acta OtoLaryngol* **114**: 105–109

Spruck CH 3d, Tsao YC, Juang DP, Yang AS, Rodeout WM 3d, Gonzalez-Zulueta M, Choi P, Lo K-W, Yu MC and Jones PA (1992) Absence of p53 gene mutations in primary nasopharyngeal carcinomas. *Cancer Res* **52**: 4787–4790

Sun Y, Hegamyer G and Colburn NH (1993) Nasopharyngeal carcinoma shows no detectable retinoblastoma susceptibility gene alterations. *Oncogene* **8**: 791–795

Sun Y, Hildesheim A, Lainer AE; Cao Y, Yao KT, Raab-Traub N and Yang CS (1995) No point mutation but decreased expression of the p16/MTS1 tumor suppressor gene in nasopharyngeal carcinomas. *Oncogene* **10**: 785–788

Too CK and Guernsey DL (1992) Increased messenger RNA levels of the antagonist thyroid hormone receptor erb A-α2 and decreased levels of erb A-α1 and erb A-β1 receptor messenger RNAs in neoplastic rodent cells. *Cancer Res* **52**: 2186–2190

Warrel RP, deThe H, Wang ZY and Degos L (1994) Acute promyelocytic leukemia. *New Eng J Med* **329**: 177–189

Zhan F, Jiang N, Cao L, Deng L, Tan G, Zhou M, Xie Y and Li G (1998) Primary study of differentially expressed cDNA sequences in cell line HNE1 of human nasopharyngeal carcinoma by cDNA representational difference analysis. *Chung Hua I Hsueh I Chuan Hsueh Tsa Chih* **15**: 341–344

 *© 2000 Cancer Research Campaign*
